# FEEDING DIFFICULTIES IN PRESCHOOL CHILDREN, PREVIOUS FEEDING PRACTICES, AND NUTRITIONAL STATUS

**DOI:** 10.1590/1984-0462/;2018;36;1;00004

**Published:** 2017-10-30

**Authors:** Hélcio de Sousa Maranhão, Renata Cunha de Aguiar, Débora Teixeira Jales de Lira, Mônica Úrsula Figuerêdo Sales, Nathalia Ávila do Nascimento Nóbrega

**Affiliations:** a Universidade Federal do Rio Grande do Norte, Natal, RN, Brasil.

**Keywords:** Eating habits, Preschool child, Breast feeding, Nutritional status, Hábitos alimentares, Pré-escolar, Aleitamento materno, Estado nutricional

## Abstract

**Objective:**

To identify the prevalence of feeding difficulties in preschoolers, its association with epidemiological factors and previous eating habits, and repercussion on nutritional status.

**Methods:**

Cross-sectional study with a questionnaire given to the mothers of 301 children aged 2-6 years enrolled in public and private kindergartens in Natal, Northeast Brazil, conducted in 2014-2015. Feeding difficulty was assessed according to Kerzner’s criteria, resulting in the profiles “highly selective intake”, “active child with small appetite”, “fear of feeding”, and “child with psychological disorder or neglected”. Association with the following independent variables was analyzed by logistic regression: breastfeeding time, age of cows’ milk and complementary feeding introduction, age range, family income, type of school, mothers’ profile (responsive or nonresponsive), and body mass index (BMI).

**Results:**

Feeding difficulty was found in 37.2% of cases, with predominance of “highly selective intake” (25.4%). It was not associated with infancy feeding practices, family income or type of school. There were no differences between the BMI Z score means for the groups with and without feeding difficulty (1.0±1.5 SD and 1.1±1.4 SD, respectively). The five-to-six age range had more occurrences (OR 1.8; 95%CI 1.1-2.9). Children of responsive mothers were less likely to have feeding difficulties (OR 0.4; 95%CI 0.2-0.8).

**Conclusions:**

Feeding difficulties were very frequent. Nutritional status was not impacted by it, and infancy eating habits were not associated with it. Responsive mothers’ profile is a protective factor against eating difficulties and reinforces the importance of behavioral factors and mother-child interaction.

## RESUMO

**Objetivo:**

Identificar a prevalência de dificuldade alimentar (DA) em pré-escolares, sua associação com fatores epidemiológicos e práticas alimentares pregressas, bem como sua repercussão sobre o estado nutricional.

**Métodos:**

Estudo transversal com aplicação de questionário às mães de 301 crianças de dois a seis anos de creches públicas e privadas em Natal, Rio Grande do Norte, em 2014 e 2015. Identificou-se DA segundo critérios de Kerzner, incluindo os perfis de “ingestão altamente seletiva”, “criança agitada com baixo apetite”, “fobia alimentar” e “criança com distúrbio psicológico ou negligenciada”. As variáveis de associação analisadas por regressão logística foram: tempo de aleitamento materno, idade de introdução de leite de vaca e da alimentação complementar, faixa etária, renda familiar, tipo de escola, perfil das mães (responsivas ou não responsivas) e índice de massa corpórea (IMC).

**Resultados:**

DA foi encontrada em 37,2% dos casos analisados, com predomínio de “ingestão altamente seletiva” (25,4%). Não houve associação entre DA e práticas alimentares na fase de lactente, renda familiar e tipo de escola. Não houve diferença entre as médias de escore Z IMC para os grupos com e sem DA (1,0±1,5DP e 1,1±1,4DP, respectivamente). A faixa etária de cinco a seis anos apresentou maior ocorrência de DA (OR 1,8; IC95% 1,1-2,9) e filhos de mães com perfil responsivo tiveram menores chances de apresentar DAs (OR 0,4; IC95% 0,2-0,8).

**Conclusões:**

DA foi de alta prevalência. Não houve repercussão sobre o estado nutricional nem associação às práticas alimentares pregressas. O perfil responsivo das mães é fator protetor para as DAs e reforça a importância da natureza comportamental e da interação mãe-filho.

## INTRODUCTION

Food difficulty (FD) is any problem that negatively affects the process of providing food or nutrients to children by parents or caregivers. This term comprises various eating disorders with different levels of severity and possibility of repercussion on nutritional status, relationship with parents, and interaction with peers.^1^ ^,^ ^2^ It is estimated that 8 to 50% of children have FD, depending on the diagnostic criteria used,^3^ and more than half of parents describe their children as selective or who eat little.^4^

Good or bad dietary practices, especially in the first thousand days of life - from gestation to two years of age -, have resonance throughout life. Although it is well established that breastmilk is sufficient as sole source of food until six months of age, a survey conducted by the Ministry of Health in 2009 showed that the average duration of exclusive breastfeeding was 1.8 months in the country.^6^ However, in recent years, strong investments and incentive, support, protection initiatives have progressively improved indicators and made Brazil a reference in breastfeeding in 2016.^7^

Proper introduction of complementary feeding after six months is an indisputable factor for the maintenance of a child’s nutritional and health status.^5^ There are reports of association between short duration of breastfeeding and early introduction of complementary food with development of selective eating in childhood, also known as picky eating.^8^ ^,^ ^9^ Shim et al.^9^ reported that exclusive breastfeeding for six months combined with introduction of complementary feeding only after this age reduces chances of the child developing a selective eating behavior between two and three years old. In contrast, Finistrella et al.^10^ found no association between duration of breastfeeding and eating phobia, defined as lack of interest in trying new food.

Likewise, there is evidence of association between sociodemographic data, especially maternal schooling, and good quality food intake.^11^ ^,^ ^12^ Family has a decisive influence on food intake self-control and on the creation of an adequate/inadequate dietary behavior pattern.^13^

Although the complaint “my child does not eat” is frequent in pediatric clinics, recognizing FD is not easy as there are few studies on the subject, lack of standardization in nomenclature for different clinical profiles, inadequate control of sociodemographic variables, and the use of retrospective data provided by parents is subjected to memory bias.^1^ ^,^ ^2^ ^,^ ^14^ ^,^ ^15^

Despite the wider knowledge about the “picky” profile, relationship between infancy feeding practices and occurrence of FD afterwards is still to be clarified. Likewise, it has not yet been established whether FD would compromise nutritional status in the long term. These findings encouraged the present study, which was aimed to identify FD in preschool children, association with epidemiological factors or previous feeding practices, and its repercussion on nutritional status.

## METHOD

Cross-sectional study carried out with a convenience sample of 301 children aged two years to six incomplete years, enrolled in four municipal nursery schools and three private schools in Natal, Rio Grande do Norte (north: 19.9%; south: 30.2%; east: 39.9%; west: 10%) between October 2014 and April 2015. Exclusion criteria were occurrence of organic diseases such as diarrhea, vomiting, asthma, red patches on skin (urticaria or eczema), food allergy, blood in stool, weight loss, frequent infections, and delayed development.^2^

After orienting the family members about the importance of the project, mothers were invited to fill a questionnaire comprised of 26 objective and easily understandable questions divided into four blocks: mother and child sociodemographic data, current eating behavior of the child (occurrence of FD), and behavioral profile of the mother before feeding the child.

Although we lack validated tools to diagnose FD, Kerzner proposed a classification, in 2009, based on clinical characteristics which were sorted in seven profiles:

1. Misinterpretation by parents;
2. High selective eating (selectivity or picky eating);
3. Very active child with small appetite;
4. Eating phobia;
5. Presence of organic disease;
6. Child with psychological disorder or neglected;
7. Crying that interferes with feeding.^1^

**Chart 1:**
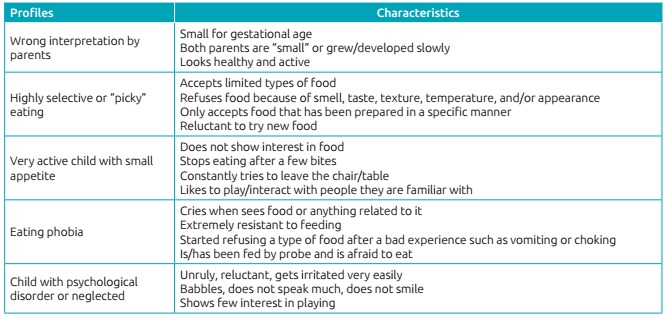
Profiles of infancy feeding difficulties according to Kerzner^1^.

This instrument was adopted for present analysis, in which characteristics of each profile were considered and presented in distinct blocks, with mothers identifying manifestations that would best represent their children’s eating behavior (Chart 1). Two of these profiles were excluded: “crying that interferes with feeding”, because it involves an age range different from of our sample, and “presence of organic disease”, as FD could result from diseases. Profiles 2, 3, 4, and 6 were analyzed in FD group, while Profile 1 was analyzed in No-FD Group. In addition, presence of FD was also evaluated in isolation, according to what mothers perceived as such.

The following feeding practices in infancy phase were retrospectively evaluated: exclusive breastfeeding time, age of cows’ milk and other food introduction. Mothers’ profiles were sorted as responsive and non-responsive, the latter comprising passive, permissive mothers.^16^ The sociodemographic data analyzed were: mother’s age; mother’s education; mother’s marital status; parentage succession; child’s age; birth weight; time of pacifier use; public or private school enrollment; and family monthly income.

Weight and height were measured by the authors of the study, previously trained for the technique standardization, with a calibrated digital portable scale (150 kg capacity and 100 g precision) and a portable anthropometer properly fixed to a wall. Nutritional status was evaluated by body mass index (BMI) for age, as this is a good, accurate, widely used indicator of body composition. Programs Anthro and Anthro plus (World Health Organization - WHO, Geneva, Switzerland) were used.^17^ ^,^ ^18^

Chi-square test was applied for categorical variables and Student’s t-test for numerical variables within the normality curve. The significance level was set at p<0.05, with Odds Ratio (OR) and 95%CI (95% confidence interval). Logistic regression model was used to adjust confounding variables for the dependent variable FD, and significant values were then used in the univariate analysis.

This study was approved by the Research Ethics Committee of *Universidade Federal do Rio Grande do Norte* (CAAE: 37228914.8.0000.5292). All participating children had their mothers or guardians signed the informed consent form.

## RESULTS

Mean age of children was 53.4 ± 13.7 months, with 54.6% of them presenting eutrophy, 19.8% risk of overweight, 13.2% overweight, 8.4% obesity, and 4% severe obesity. No child was thin. Almost half of children were exclusively breastfed until four months (49.7%), and 6.7% were never fed with breast milk.

Up to 12 months of age, 20.9% had already tried soda, 38.0% ice cream, 33.1% filled cookies, 35.3% candy/chocolate, 12.2% mortadella, 14.2% sausage, and 27.7% industrialized pastry, with increase to at least twice until 24 months of age for all percentages.

FD in children was reported by 25.1% of mothers. On the other hand, specific FD profiles were identified in 37.2% of children (Table 1).

**Table 1:** Clinical and epidemiological characteristics, infancy eating habits, and nutritional assessment of 301 preschoolers from both public and private school systems in Natal, Rio Grande do Norte.

Characteristics	n	Absolute frequency	Relative frequency (%)
Use of pacifier for more than 12 months	300	110	36.7
School category	301		
Public		188	62.5
Private		113	37.5
Feeding difficulty profiles	287		
Highly selective or “picky” eating		73	25.4
Very active child with small appetite		33	11.5
Eating phobia		1	0.3
Wrong interpretation by parents		7	2.4
No feeding difficulty		172	59.9
Exclusive breastfeeding (<6 months)	290	206	71.0
Age of cows’ milk introduction (≤6 months)	296	167	56.4
Age of food introduction (≤6 months)	297	189	63.6
Nutritional status	227		
Eutrophic		122	54.2
Risk of overweight/overweight		75	33.3
Obese/severely obese		28	12.4

Upon univariate analysis, factors associated with FD were: children aged five to six years, from private schools, who had used pacifiers for more than 12 months, family income higher than two minimum wages. However, only age range and mothers’ profile remained significant at multivariate analysis. Children aged five to six years were twice as likely to present FD. On the other hand, responsive mother profile was shown to be a protective factor (Table 2).

**Table 2:** Feeding difficulty according to age range, use of pacifier, school category, family income, and profile of mothers of 301 preschoolers from both public and private school systems in Natal, Rio Grande do Norte.

	Feeding difficulty				Univariate analysis	Multivariate analysis
	Yes		No		p-value	p-value
	n	%	n	%	OR (95%CI)	OR (95%CI)
Age range (years)						
2-4	58	32.4	121	67.6	0.02 1.79 (1.09-2.94)	0.01 1.98 (1.15-3.40)
5-6	49	46.2	57	53.8		
Use of pacifier (months)						
>12	37	12.4	72	24.2	<0.01 2.04 (1.20-3.48)	0.13 0.66 (0.38-1.13)
≤12	38	12.8	151	50.7		
School category						
Public	53	30.5	121	69.5	<0.01 2.09 (1.28-3.41)	0.14
Private	54	47.8	59	52.2		0.47 (0.18-1.27)
Monthly family income (minimum wages)						
<2	42	29.6	100	70.4	<0.01 1.94 (1.18-3.21)	0.84 0.91 (0.34-2.42)
≥2	58	45.0	71	55.0		
Profile of mothers						
Responsive	71	33.2	143	66.8	<0.01 0.45 (0.22-0.85)	<0.01 0.40 (0.22-0.73)
Not responsive	35	52.2	32	47.8		

OR: *Odds Ratio*; 95%CI: 95% confidence interval.

Feeding practices in infancy were not associated with present FD (Table 3). No significant difference was found between means of BMI Z score in FD Group (1.01 ± 1.54 SD) and in No-FD Group (1.13 ± 1.40; p = 0.13).

**Table 3:** Feeding difficulty according to infancy eating habits of 301 preschoolers from both public and private school systems in Natal, Rio Grande do Norte.

	Feeding difficulty				Chi-square test p-value
	Yes		No		
	n	%	n	%	
Exclusive breast-feeding time (months)					
≤6	72	36.2	127	63.8	0.58
>6	31	39.7	47	60.3	
Age of cows’ milk introduction (months)					
≤6	63	38.9	99	61.1	0.59
>6	44	35.8	79	64.2	
Age of food introduction (months)					
≤6	71	39.4	109	60.6	0.28
>6	34	33.0	69	67.0	

## DISCUSSION

Most of the children in this series did not receive exclusive breastfeeding as recommended by WHO,^5^ up to six months of life. This data reinforce inappropriate weaning practices and show that introduction of foods other than breast milk at early stages of life is very frequent, as pointed out by a national study.^6^ Also, excessive intake of processed products such as sausages, presumably with high sodium and sugar concentrations, in the first two years of life is alarming, as there is growing evidence of how important it is to avoid these foods in order to create healthy eating habits and, consequently, prevent chronic non-communicable diseases.

Many studies have shown the relationship of early weaning with risk of future diseases such as hypertension, dyslipidemias, metabolic syndrome, diabetes mellitus, and even some cancers,^19^ even though knowledge about the direct consequences of industrialized food intake by infants is still emergent.^20^ Thus, focus must be given to education of parents and children in order to promote healthy eating habits.

The best knowledge about FD in childhood is relevant and priority, for this is an increasingly frequent type of disorder and there is the need to perform deeper assessments of relationships between dietary, epidemiological, and clinical factors. Professionals involved in children’s health should be attentive to recognize such processes and their different profiles in order not to minimalize diagnosis,^2^ considering that ignoring them can lead to unnecessary investigations of organic processes. However, it is accepted that, for the purpose of validation, tools used to detect this disorder should be more sensible and less subjective. This gap brings limitations to known studies,^9^ ^,^ ^10^ ^,^ ^14^ ^,^ ^15^ ^,^ ^21^ ^,^ ^22^ ^,^ ^23^ although it does not devalue them in the condition of preliminary studies that point the direction for further investigations.

In 2013, Benjasuwantep et al.,^15^ used Kerzner’s criteria1 and reported prevalence of FD in 26.9% of their sample, with predominance of “highly selective or picky eating” and “very active child with small appetite” profiles, which is similar to findings of our casuistry. More national studies on this subject are still pending. In a review published in 2015, Taylor et al. described percentages of “picky eating” profile ranging from 5.6 to 50%, and this oscillation is suggested to result from different definitions and diagnostic methods.^14^

As children grow, appetite becomes more sensitive to influences from out of their households, and children’s pleasure and interest in food decreases.^24^ These factors may contribute to the higher prevalence of FD in older children. Facing that, Caton et al. suggest that the earlier exposure to different types foods improves food acceptability, as long as it takes place at an appropriate time.^25^ It is not ruled out that children may already present such complaints at an earlier moment of life given the highest prevalence resulting from cumulative effect.

The relationship between use of pacifiers for more than one year and occurrence of FD grabs attention, considering that some studies report their use as related to early weaning and sensory/motor oral alterations. Prolonged use of pacifiers would lead to maxillary alterations with open bite, a risk factor for eating disorders in children.^26^ Nonetheless, non-reproducibility of this relationship in the multivariate analysis suggests that other factors related to pacifier use, such as malocclusion of teeth, may be more decisive for the onset of this problem, although this variable was not evaluated in our study.

It is assumed that the lack of resources in developing countries for lower income groups may contribute to a broader acceptance of food put on the table, which would minimize the occurrence of FD, as demonstrated in this study. But such findings differ from those by Tharner et al.,^23^ in the Netherlands: the authors found association between food selectivity and children with lower family income. It is speculated that differences in purchasing power and social profiles in low-income households in developed and developing countries are factors that contribute with such disparity.

Greater occurrence of FD in children with controlling, indulgent or passive mothers points to the need for parents to adopt favorable responses regarding their children’s feeding, thus protecting them from the problem.^4^ Food restriction should therefore be avoided, since parents’ imposing behavior does not predict changes in children’s behavior in medium to long term^27^ and such kind of pressure decreases even more the children’s enjoyment during meals.^23^

Breastfeeding is likely to have a protective effect against picky eating behavior in childhood, since it encourages the infant to recognize flavors of food consumed by the mother.^28^ However, the literature is not consensual as to the association between FD and practices during nursing period. Such relationship may be true when observed in specific profiles such as the picky child,^8^ ^,^ ^9^ ^,^ ^10^ ^,^ ^14^ however, it is not seen when different profiles are analyzed together, as shown in our study.

As for nutritional status, absence of low weight or thinness is emphasized, since the sample was composed of children from different economic levels. Overweight, on the other hand, represented by the high percentages of overweight and obesity, becomes a worrying reality that reflects forthright nutritional transition already established in children living in the Northeast region of the country.^29^

Although there is a tendency to low weight and lower fat-free mass percentage in children with specific profiles such as fussy eater and picky eater,^28^ ^,^ ^30^ no significant differences were found as to nutritional status of children with or without FD, which supports findings by Svensson et al.^24^ and Tharner et al.^23^ Thus, one can state that further longitudinal studies are necessary so we understand the influence of eating behaviors on the development of hidden hunger, as well as repercussions on nutritional status, ranging from nutritional deficits to surpluses, including obesity.

It is assumed that a cross-sectional design imposes limitations when evaluating cause-effect relationship between the variables. The lack of longitudinal perspective also increases chances of memory bias when it comes to retrospective information about past eating practices, as these depend heavily on the memory of parents. Furthermore, children’s behavior and dietary intake were not objectively assessed through dietary records, but rather by maternal observation, as in most studies on this subject. Finally, convenience analysis has limitations, but we believe results are reliable in view of adequate sample size and data collection in public and private schools with different socioeconomic profiles, which allowed achieving specific goals of the research within a diversity.

FD is very frequent in preschoolers. Feeding practices during infancy or subsequent repercussions on nutritional status were not proven associated with it. Family income, school type, and pacifier use, despite a preliminary association with FD, were shown to be confounding variables only, and were not relevant in the final analysis. Among preschoolers, the 5-6 age range holds the highest percentages of this disorder. Responsive mother profile as to infancy feeding is, however, a protective factor for FD and underlines the importance of maternal behavior and mother-child interaction over eating difficulties.
